# Evolution of koinobiont parasitoid host regulation and consequences for indirect plant defence

**DOI:** 10.1007/s10682-022-10180-x

**Published:** 2022-05-09

**Authors:** Maximilien A. C. Cuny, Erik H. Poelman

**Affiliations:** Laboratory of Entomology, Wageningen University, P.O. Box 16, 6700 AA Wageningen, The Netherlands

**Keywords:** Natural enemies, Host manipulation, Parasitism, Host growth, Symbiont, Tritrophic interaction

## Abstract

Tritrophic interactions among plants, herbivorous insects and their parasitoids have been well studied in the past four decades. Recently, a new angle has been uncovered: koinobiont parasitoids, that allow their host to keep feeding on the plant for a certain amount of time after parasitism, indirectly alter plant responses against herbivory via the many physiological changes induced in their herbivorous hosts. By affecting plant responses, parasitoids may indirectly affect the whole community of insects interacting with plants induced by parasitized herbivores and have extended effects on plant fitness. These important findings have renewed research interests on parasitoid manipulation of their host development. Parasitoids typically arrest their host development before the last instar, resulting in a lower final weight compared to unparasitized hosts. Yet, some parasitoids prolong their host development, leading to larger herbivores that consume more plant material than unparasitized ones. Furthermore, parasitoid host regulation is plastic and one parasitoid species may arrest or promote its host growth depending on the number of eggs laid, host developmental stage and species as well as environmental conditions. The consequences of plasticity in parasitoid host regulation for plant–insect interactions have received very little attention over the last two decades, particularly concerning parasitoids that promote their host growth. In this review, we first synthesize the mechanisms used by parasitoids to regulate host growth and food consumption. Then, we identify the evolutionary and environmental factors that influence the direction of parasitoid host regulation in terms of arrestment or promotion of host growth. In addition, we discuss the implication of different host regulation types for the parasitoid’s role as agent of plant indirect defence. Finally, we argue that the recent research interests about parasitoid plant-mediated interactions would strongly benefit from revival of research on the mechanisms, ecology and evolution of host regulation in parasitoids.

## Introduction

Parasitoids are typically small wasps that lay their eggs inside (endoparasitoids) or outside (ectoparasitoids) a host that serves as food source for the entire larval stage of the parasitoids offspring. They are important model organisms in the study of behavioural and evolutionary ecology that are ubiquitous in many natural and agricultural ecosystems: virtually all immature insect can be parasitized by at least one parasitoid species (Godfray 1994).

Recently, studies have shed light on a novel ecological phenomenon in tritrophic interactions: koinobiont parasitoids indirectly affect plant responses to herbivory, which, in turn, alter the attraction (Cusumano et al. 2018; Zhu et al. 2018) and performance (Tan et al. 2018, 2019) of subsequent herbivores. Plant recognition of parasitized herbivores is altered because of the important regulation imposed by parasitoids on their host physiology (Cusumano and Volkoff 2021). Such host manipulation is achieved via several types of factors injected by parasitoids in the host along with eggs, such as symbiotic viruses and venom (Vinson and Iwantsch 1980). This exciting new angle of tritrophic interactions is receiving increasing attention (Cusumano et al. 2021; Dicke et al. 2020; Poelman and Cusumano 2021; Tan et al. 2020; Wang et al. 2021) and has renewed research interests for parasitoid host regulation. Host regulation has been understudied over the last two decades, and many areas are still not fully understood, particularly concerning the mechanisms, plasticity and ecological consequences of parasitoid promotion of host growth.

Parasitoids are generally divided into two categories of host usage strategies: idiobiosis and koinobiosis (Harvey 2005; Mackauer and Sequeira 1993). In the first strategy, parasitoids either paralyze their host, preventing them from moving and feeding, or parasitize sessile host stages (e.g. eggs or pupae). In the second strategy, koinobiont parasitoids develop in a growing host that keeps on moving and feeding for a significant period of the development of the parasitoid larva. Idiobionts are generally ectoparasitoids with a wide host range while koinobionts are typically endoparasitoids and considered to be more specialized (Santos and Quicke 2011). The common ancestor of parasitoids was an idiobiont ectoparasitoid that attacked concealed hosts (Pennacchio and Strand 2006). Koinobiosis as adaptation allows parasitoids to lay their eggs in hosts at an earlier stage of development and without the risk of the parasitoid eggs or larvae becoming disconnected from their host (Mackauer et al. 1997; Strand 2000). On the other hand, the continuous development of the host can be a constraint when the host environment becomes more hostile during larval development. Koinobiont parasitoids overcome this constraint by regulating their host development for their own benefit (Beckage and Gelman 2004). The degree of host manipulation in parasitoids ranges from ‘conformers’ that are adapted to the normal development of their host with limited host manipulation to parasitoids that are strong ‘regulators’ that induce significant changes in their host development (Lawrence 1986). However, in several species, parasitoid larvae are plastic in the level of host regulation depending on factors such as host age or species (Harvey et al. 1999; Harvey and Malcicka 2016; Mackauer and Sequeira 1993).

In many documented cases of host regulation, koinobiont parasitoids prematurely stop the development of their herbivorous host which results in a reduction of their final size and host food consumption (Table 1; Beckage and Gelman 2004; Harvey 1996; Varley and Butler 1933). Alternatively, some koinobiont parasitoids extend their host development time (for example, by inducing a supernumerary larval instar) or increase their feeding rate, resulting in a higher final host weight compared to unparasitized hosts (Table 2; Ode 2006). Furthermore, some parasitoids exert plasticity in the direction of their host regulation and are able to arrest or prolong their host development according to environmental conditions (Harvey 1996). Several factors may explain why parasitoids evolved such opposite strategies of host regulation, such as reduction of their host predation risk or parasitoid resource needs (Fritz 1982; Harvey 2005; Pennacchio and Strand 2006).

**Table 1:** Non-exhaustive review of the literature reporting a decrease in parasitized herbivore weight or food consumption compared to unparasitized herbivores. Decrease of herbivore growth has been found in Hemipteran and Lepidopteran hosts and may yield hosts that are up to 97% smaller than unparasitized hosts

Parasitoid species	Gregarious/Solitary parasitoid	Host species	Host order	Effect of parasitism compared to healthy hosts (%)	Measurement	Reference
*Alabagrus texanus*	Solitary	*Herpetogramma theseusalis*	Lepidoptera	− 40	Mean weight	Morse and Chapman (Morse and Chapman 2015)
*Aphidius ervi*	Solitary	*Acyrthosiphon pisum*	Hemiptera	− 40	Maximum weight	Sequeira and Mackauer (1992)
*Campoletis grioti*	Solitary	*Spodoptera frugiperda*	Lepidoptera	− 96	Maximum weight	Ashley (1983)
*Campoletis sonorensis*	Solitary	*Spodoptera frugiperda*	Lepidoptera	− 83	Maximum weight	Isenhour et al. (1988)
*Campoletis sonorensis*	Solitary	*Helicoverpa virescens*	Lepidoptera	− 69	Maximum weight	Vinson (1972)
*Cardiochiles nigriceps*	Solitary	*Helicoverpa virescens*	Lepidoptera	− 75	Food consumption	Guillot and Vinson (1973)
*Chelonus inanitus*	Solitary	*Spodoptera littoralis*	Lepidoptera	− 86	Maximum weight	Morales et al. (2007)
*Chelonus insularis*	Solitary	*Spodoptera frugiperda*	Lepidoptera	− 81	Maximum weight	Ashley (1983)
*Cotesia congregata*	Gregarious	*Manduca sexta*	Lepidoptera	− 39	Maximum weight	Alleyne and Beckage (1997)
*Cotesia congregata*	Gregarious	*Manduca sexta*	Lepidoptera	− 8	Maximum weight	Moore et al. (2020)
*Cotesia flavipes*	Gregarious	*Diatraea saccharalis*	Lepidoptera	− 27	Food consumption	Rossi (2014)
*Cotesia marginiventris*	Solitary	*Spodoptera frugiperda*	Lepidoptera	− 97	Maximum weight	Ashley (1983)
*Cotesia marginiventris*	Solitary	*Spodoptera litura*	Lepidoptera	− 88	Maximum weight	Jalali and Ballal (1988)
*Cotesia marginiventris / Campoletis sonorensis*	Solitary	*Spodoptera littoralis*	Lepidoptera	− 97	Maximum weight	Hoballah and Turlings (2001)
*Cotesia rubecula*	Solitary	*Pieris rapae*	Lepidoptera	− 88	Food consumption	Parker and Pinnell (1973)
*Cotesia rubecula*	Solitary	*Pieris rapae*	Lepidoptera	− 77	Food consumption	Rahman (1970)
*Cotesia rubecula*	Solitary	*Pieris rapae*	Lepidoptera	− 87	Maximum weight	Harvey et al. (1999)
*Cotesia rubecula*	Solitary	*Pieris brassicae*	Lepidoptera	− 97	Maximum weight	Harvey et al. (1999)
*Eiphosoma vitticole*	Solitary	*Spodoptera frugiperda*	Lepidoptera	− 75	Maximum weight	Ashley (1983)
*Hyposoter didymator*	Solitary	*Helicoverpa armigera*	Lepidoptera	− 88	Maximum weight	Mironidis and Savopoulou-Soultani (2009)
*Hyposoter didymator*	Solitary	*Spodoptera littoralis*	Lepidoptera	− 93	Maximum weight	Morales et al. (2007)
*Hyposoter ebeninus*	Solitary	*Pieris rapae*	Lepidoptera	− 77	Maximum weight	Harvey et al. (2010a)
*Hyposoter ebeninus*	Solitary	*Pieris brassicae*	Lepidoptera	− 76	Maximum weight	Harvey et al. (2010a)
*Hyposoter exigua*	Solitary	*Trichoplusia ni*	Lepidoptera	− 82	Maximum weight	Thompson (1982)
*Meteorus pulchricornis*	Solitary	*Mythimna separata*	Lepidoptera	− 97	Maximum weight	Harvey et al. (2010b)
*Microplitis croceipes*	Solitary	*Helicoverpa zea*	Lepidoptera	− 79	Maximum weight	Jones and Lewis (1971)
*Microplitis demolitor*	Solitary	*Helicoverpa virescens*	Lepidoptera	− 94	Maximum weight	Strand et al. (1988)
*Microplitis similis*	Solitary	*Spodoptera exigua*	Lepidoptera	− 38	Maximum weight	Li et al. (2015)
*Microplitis similis*	Solitary	*Spodoptera litura*	Lepidoptera	− 76	Maximum weight	Li et al. (2015)
*Microplitis tristis*	Gregarious	*Hadena bicruris*	Lepidoptera	− 51	Maximum weight	Elzinga et al. (2003)
*Microplitis tuberculifer*	Solitary	*Mythimna separata*	Lepidoptera	− 96	Maximum weight	Chu et al. (2014)
*Rogas laphygmae*	Solitary	*Spodoptera frugiperda*	Lepidoptera	− 85	Maximum weight	Isenhour et al. (1988)
*Venturia canescens*	Solitary	*Galleria mellonella*	Lepidoptera	− 84	Maximum weight	Harvey (1996)
*Venturia canescens*	Solitary	*Anagasta kuehniella*	Lepidoptera	− 65	Maximum weight	Harvey (1996)

**Table 2:** Review of the literature reporting an increase in parasitized herbivore weight, size, developmental time or feeding behaviour. Increase of herbivore growth has been found in Hemipteran and Lepidopteran hosts and may yield hosts that are up to 81% larger than unparasitized hosts. Increase of host growth has received little attention on research agendas of the last two decades

Parasitoid species	Gregarious / Solitary parasitoid	Host species	Host order	Effect of parasitism compared to healthy hosts (%)	Measurement	Comment	Reference
*Aphidius ervi*	Solitary	*Acyrthosiphon pisum*	Hemiptera	+ 35	Host food consumption	Lower food assimilation	Cloutier and Mackauer (1979)
*Aphidius ervi*	Solitary	*Acyrthosiphon pisum*	Hemiptera	+ 53	Host food consumption	Superparasitism	Cloutier and Mackauer (1980)
*Copidomopsis nacoleiae*	Gregarious	*Marasmia patnalis*	Lepidoptera	+ 33	Leaf consumption	–	Arida et al. (1989)
*Copidosoma bakeri*	Gregarious	*Euxoa auxiliaris*	Lepidoptera	+ 65	Maximum weight	Longer development time	Byers et al. (1993)
*Copidosoma floridanum*	Gregarious	*Trichoplusia ni*	Lepidoptera	+ 50	Induction of plant glucosinolates	Increased feeding	Ode et al. (2016)
*Copidosoma floridanum*	Gregarious	*Trichoplusia ni*	Lepidoptera	+ 41	Weight	–	Strand (1989)
*Copidosoma sosares*	Gregarious	*Depressaria pastinacella*	Lepidoptera	+ 55	Mean weight	–	McGovern et al. (2006)
*Copidosoma truncatellum*	Gregarious	*Trichoplusia ni*	Lepidoptera	+ 30	Maximum weight	Longer development time	Hunter and Stoner (1975)
*Cotesia congregata*	Gregarious	*Manduca sexta*	Lepidoptera	+ 214	5th instar duration	–	Beckage and Riddiford (1983)
*Cotesia congregata*	Gregarious	*Manduca sexta*	Lepidoptera	+ 24	Head capsule size	Supernumerary larval instars	Beckage and Riddiford (1978)
*Cotesia congregata*	Gregarious	*Manduca sexta*	Lepidoptera	+ 50	Weight	High amount of wasp PDV	Dushay and Beckage (1993)
*Cotesia congregata*	Gregarious	*Manduca sexta*	Lepidoptera	+ 26	Weight	Injection of PDV + Venom	Reed and Beckage (1997)
*Cotesia glomerata*	Gregarious	*Pieris brassicae*	Lepidoptera	+ 81	Final larval weight	Heavily parasitized	Führer and Keja (1976)
*Cotesia glomerata*	Gregarious	*Pieris brassicae*	Lepidoptera	+ 25	Leaf consumption	Superparasitism	Gu et al. (2003)
*Cotesia glomerata*	Gregarious	*Pieris brassicae*	Lepidoptera	+ 60	Maximum weight	Superparasitism	Hasan and Ansari (2012)
*Cotesia glomerata*	Gregarious	*Pieris rapae*	Lepidoptera	+ 16	Leaf consumption	Longer development time	Rahman (1970)
*Cotesia glomerata*	Gregarious	*Pieris rapae*	Lepidoptera	+ 30	Leaf consumption	Longer development time	Parker and Pinnell (1973)
*Cotesia glomerata*	Gregarious	*Pieris rapae*	Lepidoptera	+ 8	Weight	–	Slansky (1978)
*Cotesia glomerata*	Gregarious	*Pieris rapae*	Lepidoptera	+ 64	Maximum weight	Heavily parasitized	Harvey (2000)
*Cotesia kariyai*	Gregarious	*Pseudaletia separata*	Lepidoptera	+ 50	Development time	Host parasitized at late instar	Sato et al. (1986)
*Cotesia plutellae*	Solitary	*Plutella xylostella*	Lepidoptera	+ 43	Fresh leaf consumption	Longer development time	Shi et al. (2002)
*Eucelatoria sp*	Gregarious	*Helicoverpa virescens*	Lepidoptera	+ 15	Host food consumption	Host parasitized at last instar	Brewer and King (1980)
*Euplectrus platyhypenae and E. comstockii*	Gregarious	*Helicoverpa virescens*	Lepidoptera	+ 7	Maximum weight	Host parasitized at last instar	Coudron et al. (1997)
*Meteorus pulchricornis*	Solitary	*Plutella xylostella*	Lepidoptera	+ 30	Maximum weight	Small host species	Harvey et al. (2010a, b)
*Psyllaephagus baccharidis*	Solitary	*Baccharopelma dracunculifoliae*	Hemiptera	+ 41	Gall volume	Higher host feeding	Espìrito-Santo et al. (2004)
*Pteromalus albipennis*	Solitary	*Tephritis femoralis*	Diptera	+ 25	Weight	Longer development time	Xi et al. (2015)

When parasitism results in a premature arrestment of the herbivorous host development, it often reduces the host plant consumption and can have a beneficial effect on plant fitness (Bustos-Segura et al. 2019; Gols et al. 2015; Gómez and Zamora 1994; Hoballah and Turlings 2001; van Loon et al. 2000). Consequently, plants are hypothesized to have evolved several traits to increase parasitoid attraction (e.g. extrafloral nectar, volatiles) as part of plant indirect defence strategies (Gols 2014; Pearse et al. 2020; Schuman et al. 2012). However, these traits may also attract parasitoids that do not reduce or even enhance plant fitness costs of feeding by the herbivore host (Cuny et al. 2021).

Here, we first review the physiological and molecular traits used by koinobiont parasitoids in order to regulate their host development and feeding behaviour. Then, we identify the evolutionary and ecological factors that may be responsible for whether parasitoids increase or decrease their host weight and plant consumption. Furthermore, we discuss the evolutionary implications of koinobiont parasitoid host regulation for plant indirect defence strategies. Finally, we argue that indirect plant-parasitoid interactions can only be deciphered with a full understanding of how parasitoids regulate their host development.

## Mechanisms of koinobiont parasitoid host developmental regulation and feeding behaviour

Parasitoids significantly change the physiology of their host in order to render it more suitable for an optimal development of the parasitoid larva(e) (Beckage and Gelman 2004; Vinson and Iwantsch 1980). Host regulation is often a concerted process directed by the parasitoid larva itself and factors that parasitoids inject along with the eggs into the host, such as endogenous viruses, venom, and teratocytes. Although these factors may be injected to suppress the immune system of the host (Vinson 1990), we here focus on their role in regulation of the host development and feeding behaviour.

- Polydnaviruses (PDVs)
  Many parasitoids harbour endogenous viruses from the family of the Polydnaviridae that reproduce in the calyx of adult wasps and are injected inside the host during oviposition (Rotheram 1967; Stoltz et al. 1984; Strand and Burke 2019). The association with viruses arose in two separate lineages of parasitoids belonging to the Braconidae and Ichneumonidae families (Strand and Burke 2015). The polydnaviruses (PDVs) are therefore divided into two groups: bracoviruses and ichnoviruses. Once released into the host, PDVs infect the host cells and discharge their DNA into the nuclei. As a consequence, the host cells integrate virus DNA segments into their genome and start producing PDV gene products such as protein tyrosine phosphatases (PTPs, (Pruijssers and Strand 2007)). These products are released into the host and have a strong effect on its immune system, but also on the regulation of host growth (Strand and Burke 2014).
  PDV host regulation is mainly achieved through alterations of the host hormonal levels (e.g. juvenile hormone and ecdysteroid) or neuropeptides that are controlling metamorphosis, pupation or feeding behaviour (Dushay and Beckage 1993; Ignesti 2018; Shi et al. 2015). PDVs can also induce metabolic changes in the host, such as hyperglycemia, resulting in an arrestment of the feeding behaviour of the host and a reduction in weight (Pruijssers et al. 2009). In most of the reported cases, PDVs reduce the size, inhibit moulting and cause an early arrestment of the development and feeding of their host (Dorémus et al. 2014; Strand and Burke 2014). Although less well documented, PDVs can also prolong the host development time and increase its final weight (Beckage et al. 1994; Doucet and Cusson 1996). For example, *Manduca sexta* larvae injected with calyx fluid derived from parasitoids had a longer developmental time and a higher weight than hosts injected with parasitoid eggs that were parted of the calyx fluid (Dushay and Beckage 1993).

(b) Venom
  While parasitizing their host, koinobiont parasitoids also inject non-paralysing venom, a complex mixture mainly composed of enzymes with diverse functions (Asgari and Rivers 2011; Poirié et al. 2014). It is produced in the parasitoid venom gland and stored in its reservoir. Venom injected by koinobiont endoparasitoids typically plays an important immunosuppressive role (Asgari and Rivers 2011; Moreau and Asgari 2015), but also affects their host development (Digilio et al. 1998). For koinobiont parasitoids that harbour PDVs, venom also plays a synergistic role in the support of the PDVs functions. When PDVs are experimentally injected into the host without venom, host development is prolonged because ecdysteroid disruption is either reduced or not observed anymore (Digilio et al. 1998; Strand and Dover 1991; Tanaka 1987; Tanaka and Vinson 1991). Similarly, herbivorous larvae parasitized by parasitoids lacking the poison gland lived longer and consumed more food than normally parasitized hosts (Guillot and Vinson 1973). In some cases, venom can even be mandatory for the survival of PDVs in the host (Stoltz et al. 1988).

(c) Teratocytes
  Some parasitoid species from two families (Braconidae and Platygastroidea) inject eggs that have a specialized membrane that differentiates into autonomous cells (so called: "teratocytes", (Dahlman 1990; Strand 2014)) that are released into the host haemolymph when the parasitoid egg hatches (Pedata et al. 2003; Vinson 1970). Teratocytes play an important role in the arrestment of the host growth by the production of proteins and miRNAs that interfere with host hormones (e.g. juvenile hormones or ecdysteroids) that control its growth and metamorphosis (Falabella et al. 2000; Joiner et al. 1973; Wang et al. 2018; Zhang et al. 1992, 1997). In addition, teratocytes inhibit host protein synthesis which has negative consequences for the host growth (Dahlman et al. 2003). However, teratocytes injected alone in unparasitized hosts may prolong host development time and feeding behaviour compared to unparasitized hosts (Adamo et al. 1997). In general, the study of teratocytes has lagged behind other host regulating parasitoid factors such as PDVs and venom (Strand 2014).

(d) Parasitoid larvae
  The parasitoid larva itself can affect its host development via the production of several secretory products such as proteins, saliva or hormones. Parasitoid larvae can act on the endocrinal system of their host via the release of hormones into the haemolymph of their host, such as ecdysteroids and juvenile hormone, inducing a premature host metamorphosis or developmental arrestment (Brown et al. 1993; Cole et al. 2002; Gelman et al. 1999). They also release proteins (Vinson et al. 1994) that play a role in the control of host development (Hochuli et al. 1999). Proteins present in the saliva of some ectoparasitoid larvae allow them to kill their host just before parasitoid pupation (Nakamatsu and Tanaka 2004). For the gregarious endoparasitoid *Cotesia congregata*, when only the parasitoid larvae are injected in an unparasitized *Manduca sexta* (without venom or PDVs), the host may stop feeding after the larvae emerged, similar to naturally parasitized hosts, suggesting that the larvae are responsible for this arrestment of feeding behaviour (Adamo et al. 1997). This could be caused by an over-activation of the host immunity response (Adamo et al. 2016).

## Evolutionary and ecological factors promoting parasitoid arrestment or increase in host development

**Fig. 1:**
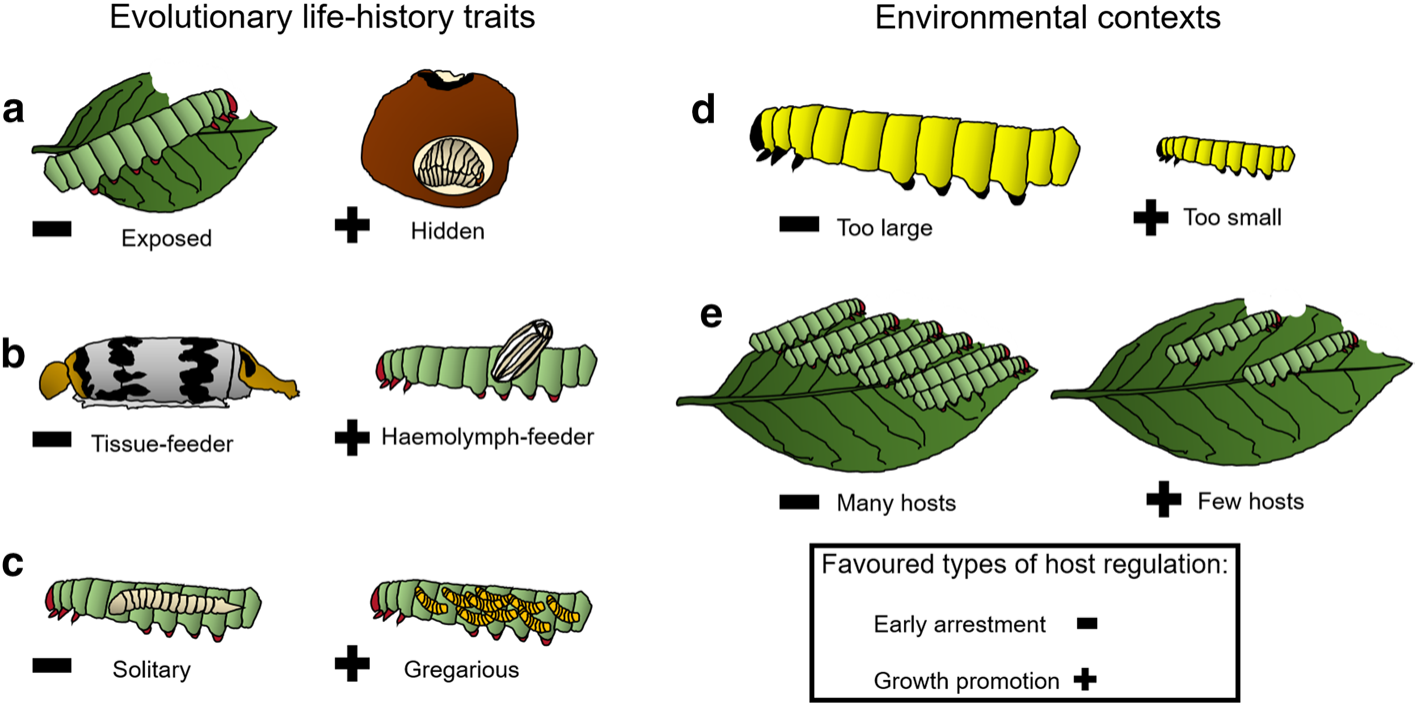
Evolutionary and environmental factors influencing the outcome of parasitoid host regulation toward an early arrestment of the host development or toward a promotion of its growth. **a**: host feeding ecology, **b**: parasitoid host-utilization strategies, **c**: parasitoid developmental strategies, **d**: host quality and **e**: host availability

^1993^). However, koinobiont parasitoids often face trade-offs between the optimisation of offspring size and development time, and other important ecophysiological factors such as host mobility and continuous development (Harvey 2005).

Whether parasitoids increase or decrease their host weight and food consumption depends on several parameters (Fig. 1). At the evolutionary scale, host feeding ecology as well as parasitoid life-history traits play an important role in shaping the type of host regulation. While at the ecological scale, parasitoid resource need for an optimal development is a good predictor for host arrestment or prolongation.

- Evolutionary factors

The type of host regulation strategy increases or decreases the time that parasitoids spend developing in their host, which can increase or decrease the host exposure to predators. This is particularly important for the fitness of parasitoids developing in an exposed host with high risks of predation, which would result in the death of the parasitoid larva(e) (Fritz 1982). Therefore, it is hypothesized that parasitoids have evolved host regulation strategies that arrest the development of their host early in their development (e.g. earlier moulting or pupation) in order to minimize the predation risks of exposed hosts. For example, parasitoid larvae that develop in exposed hosts tend to favour a faster developmental time, at the expense of a smaller final weight, and therefore arrest the development of their host earlier (Harvey and Strand 2002). On the other hand, parasitoids that develop in a concealed host with a lower risk of predation tend to favour host regulation strategies that increase host developmental time, total food consumption and final weight compared to their exposed counterparts (Harvey and Strand 2002).

The evolution of parasitoid host regulation is also constrained by the feeding ecology of parasitoid larvae. Koinobiont endoparasitoids can be categorized in two larval host-utilization strategies: tissue and haemolymph feeders (Harvey et al. 2000; Harvey and Gols 2018). Tissue feeders typically consume their host almost entirely in order to pupate. As a consequence, if the host grows too much, the parasitoid can be forced to overeat or can be trapped inside and die (Harvey 1996, 2005). Hence, we can hypothesize that for parasitoids feeding on host tissue, there is an evolutionary advantage to arrest the host development below a threshold, resulting in a development time of parasitized hosts that is usually shorter than unparasitized ones. On the other hand, parasitoids that feed on haemolymph and fat bodies typically pupate outside of their host, which they do not have to entirely consume (Harvey and Strand 2002). They are not constrained by a short host developmental time and they can allow their host to grow until the last instar. They even sometimes extend the host developmental time with a supernumerary instar which significantly increase its final weight (Table 2, Harvey and Malcicka 2016). Therefore, the capacity of feeding on the host haemolymph could have favoured host regulation strategies that stimulate host growth.

Finally, the evolution of parasitoid host regulation type is affected by their life-history strategies (i.e. solitary and gregarious (Godfray 1994)) and the amount of resource needed to complete development. Solitary parasitoids typically lay only one egg in their host. In contrast, gregarious parasitoids lay several eggs in the same host where they can all complete their development if sufficient resource is provided by the parasitized host. Gregarious parasitoid larvae need significantly more resources than their solitary counterparts. Consequently, they often prolong their host development in order to increase the amount of resource available, while solitary parasitoids often arrest their host development prematurely. All known gregarious parasitoids are haemolymph feeders, suggesting that this strategy is particularly well adapted for the high resource needs of gregarious parasitoids. Therefore, it can be hypothesized that the ability to feed on haemolymph has favoured the transition from solitary parasitoid development to gregariousness (Strand 2000), increasing parasitoid resource needs and favouring the evolution toward host growth promotion. Competition for limiting host supplies could also be an important factor favouring the evolution from solitary to gregariousness (Mackauer and Chow 2015).

(b) Environmental factors

Parasitoid host regulation can be plastic: the intensity in decreasing or increasing their host weight can greatly vary according to environmental factors linked with parasitoid resource needs. Furthermore, in different ecological contexts, parasitoids from the same species can exert either a decrease or an increase of their hosts weight.

Parasitoids that typically reduce their final host weight tend to have a stronger host regulation when developing in large hosts. Consequently, in an environment where only large hosts can be found (either large species or late instars), the reduction in host weight due to parasitism will be stronger. This is particularly the case for tissue-feeder parasitoids that entirely consume their host in order to pupate, as having too much resources can be particularly detrimental (Harvey 1996, 2005). This is important when parasitizing a host at a late instar (Harvey et al. 1994), or when parasitizing a large host species. For example, the parasitoid *Venturia canescens* has a stronger effect on the arrestment of its host development when developing in a large host species (*Galleria mellonella*) compared to a smaller one (*Anagasta kuehniella*) (Harvey 1996).

The increase of parasitized host weight tends to be stronger when the amount of resources available in the host is not sufficient for an optimal development of the parasitoid larva(e). This is particularly the case for gregarious parasitoids, as several larvae are developing in the same host and therefore the amount of resources needed is higher compared to their solitary counterparts (Table 2). Importantly, the number of parasitoid larvae developing on/in the same host greatly influences the intensity of the host developmental regulation (Harvey 2000). The higher the number of parasitoid larvae sharing the same host, the more they will regulate the host development to increase its final weight (Smallegange et al. 2008). As a consequence, all the environmental conditions that favour a higher number of gregarious parasitoid larvae developing in the same host will also favour a stronger host regulation toward a higher host plant consumption and final weight. Gregarious parasitoid clutch size usually increases according to the size of the host at parasitism (Sato et al. 1986). Additionally, hosts can also be superparasitized (i.e. laying eggs in a host that is already parasitized by a conspecific parasitoid), leading to very high numbers of parasitoid larvae in the same host and an even stronger host regulation (Table 2; Gu et al. 2003). Several environmental factors can influence the probability of superparasitism (or self-superparasitism when the same female parasitizes the same host several times), such as the amount of hosts available in the environment, the competition with other parasitoids (when superparasitism increases the probability to survive larval competition) and the necessity to exhaust the host immune system (Mackauer et al. 1992; Rosenheim and Hongkham 1996). It has to be noted that superparasitism by solitary parasitoids can also lead to an increased host food consumption (Cloutier and Mackauer 1979, 1980).

Finally, depending on the host species availability in a given environment, the same parasitoid species can either arrest or increase its host development in order to match resources requirement for the developing parasitoid larva(e). For example, *Meteorus pulchricornis*, a solitary parasitoid that feeds on its host haemolymph, strongly reduces its host development compared to unparasitized hosts when developing in a large host species (*Mythimma separata*) (Harvey et al. 2010b). Interestingly, when the same parasitoid developed in a very small host species (*Plutella xylostella*), with likely not enough resources for an optimal parasitoid larval development, host maximum weight was increased by 30% compared to an unparasitized host. Similarly, the gregarious endoparasitoids *Cotesia glomerata* either reduce or increase their host final weight when developing in a big (*Pieris brassicae*) or small (*P. rapae*) host, respectively (Harvey 2000).

## Implications of the type of host regulation for plant-parasitoid interaction

Depending on the direction of host regulation, parasitoids can have either a beneficial, neutral or negative effect on plant performance. This may have important consequences for the role of parasitoids as part of plant indirect defence.

In many cases, koinobiont parasitoid host regulation leads to a reduction of the host final weight compared to unparasitized hosts, especially when parasitoids are tissue-feeders and solitary (Table 1; Beckage and Gelman 2004; Vinson and Iwantsch 1980). In this case, parasitism usually reduces herbivory, which can in turn have a positive effect on plant fitness (Dicke and van Loon 2000; Gols et al. 2015; Gómez and Zamora 1994; Hoballah and Turlings 2001). From this point of view, the production of herbivore-induced plant volatiles that attract koinobiont parasitoids toward the plant under attack could be regarded as part of plant indirect defence strategy (Gols 2014; Pearse et al. 2020; Schuman et al. 2012). Considering that parasitoids benefit from the emission of HIPVs that allow them to find their host and increase their fitness (Turlings et al. 1990), plant-parasitoid interactions could even be classified as mutualism (van Loon et al. 2000). The adaptiveness of HIPVs to attract koinobiont parasitoids is still subject to debate (Turlings and Erb 2018). However, when the attracted natural enemies are predators that eat their prey, the advantage of HIPV emission for plant fitness is clearer (Pearse et al. 2020). This leads to the hypothesis that HIPVs are adaptively produced to attract natural enemies (among other functions, Hare 2011; Heil 2014), but it is likely that in several environments, koinobiont parasitoids are not the ideal natural enemies for a plant to attract (Cuny et al. 2021). An alternative hypothesis is that HIPVs are a by-product of plant responses to herbivore attack and parasitoids evolved to exploit these cues in host searching, without a fitness benefit to the plant.

Some koinobiont parasitoids regulate their host in a way that they increase their final weight as well as the amount of plant tissue consumed, compared to unparasitized herbivores (Ode 2006, Table 2). This can even translate into a negative effect on plant fitness (Xi et al. 2015). Therefore, parasitoids that promote their host growth should not be recruited by plants as they do not deliver indirect defence. Yet, when HIPVs are released in the environment, the emitter plant has virtually no control on the receiver species (Kessler and Heil 2011; van der Meijden and Klinkhamer 2000). This may result in the attraction of koinobiont parasitoids that have negative effects on plants due to their host regulation type (Coleman et al. 1999; Kaplan 2012). The context dependency of direction of host regulation by some parasitoids further increases the unreliability in recruiting parasitoids as indirect defence against herbivores and may sometimes turn into an antagonistic relationship.

For a plant trait to be positively selected via natural selection, the main factor to consider is the final overall fitness gain. Therefore, if on average a plant has a net fitness gain when attracting koinobiont parasitoids with HIPVs, this trait will be positively selected via natural selection, even if the plant also interacts with parasitoids that have a negative effect. This raises the following questions: (1) what is the ratio of parasitoids that reduce their host plant consumption versus parasitoids that increase it in natural environments?, (2) do they have the same effect size on plant damage? and (3) does variation in plant damage always translate into an effect on plant fitness? First, it seems that solitary and tissue-feeder parasitoids are more prevalent than gregarious and haemolymph-feeders, probably because haemolymph feeding and gregariousness are relatively recent adaptations (Harvey et al. 2000; Hoballah et al. 2004; Mayhew 1998). Assuming that in general solitary and tissue-feeder parasitoids have more chance to reduce plant damage than haemolymph-feeders and gregarious parasitoids (but see Gols et al. 2015; Harvey et al. 2000, 2010b; Xi et al. 2015), it can be hypothesized that there are more parasitoid species that tend to reduce plant damage (although this pattern could greatly vary according to the environment). Second, if we compare the size of effect of host regulation by parasitoids that increase or decrease their host weight (Tables 1, 2; Hoballah et al. 2004), it seems that, in general, the reduction of parasitized host weight is more important than the increase (approximately − 80% versus +40%, respectively). Thirdly, herbivory does not always translate into a negative effect on plant fitness: some plants can tolerate herbivore damage in order to maintain their fitness (Strauss and Agrawal 1999; Simms 2000). In such plants, variation in herbivory due to parasitism is likely to have no effect on plant fitness, although this has never been tested.

Moreover, it is also very important to consider long-term (i.e. multigenerational) effects of herbivore population reduction by parasitoids on plant fitness (Ode 2006; Pearse et al. 2020). Indeed, even though parasitoids may not always reduce plant damage, or may even increase it, they virtually all ultimately kill their host and reduce herbivore populations (Price et al. 1980). As a consequence, in a context with long-lived plants that suffer from several generations of herbivores, parasitoids have the potential to negatively affect their host population size, and to locally relieve plants from herbivory. This could be particularly visible when the migration of herbivore progeny is negligible (van der Meijden and Klinkhamer 2000). As a consequence, it can be hypothesized that even if the direct effect of parasitism is an increase of plant damage, this negative effect could be entirely compensated by the local reduction in the host population size during the following generations of herbivores.

Furthermore, for some herbivorous hosts such as caterpillars from the *Pieris* genera, parasitism may be predominantly by gregarious parasitoids. In such specific cases, there are higher chances that the overall consequences of parasitoid attraction could be negative for plant fitness. This may result in a local selection against plant volatile production followed by a local reduction in plant volatile emission (Kergunteuil et al. 2019; Kessler and Heil 2011; Schuman et al. 2012). Such local selection of parasitoids on plant defensive traits has received very little attention so far (Ode 2006, 2019).

## Conclusion and future directions

Over the last two decades, the significance of parasitoid host manipulation has been extended to indirect plant-mediated species interactions that are initiated by parasitized herbivores. We argue that the identification of an extended phenotype of parasitoid host manipulation to plant–insect interactions requires deep understanding of the changes in parasitized herbivores.

First, we should explore whether parasitoids that arrest host growth differ in their impact on plant responses to parasitized herbivores compared to parasitoids that prolong host development. Although some studies compared plant induced responses to hosts parasitized by a solitary parasitoid that arrest growth with a gregarious parasitoid that promotes development (Poelman et al. 2011a, b, 2012), these studies could not provide causal evidence for host development as driving factor for the extended phenotype of parasitoids on plant–insect interactions. This is primarily caused by under sampling of gregarious parasitoid species for these interactions. We propose that superparasitizing hosts to create variation in host development from arrestment to prolongation should be used to provide direct evidence for the importance of host development in determining induced plant responses to parasitized hosts.

Second, parasitoid host manipulation affects plant-mediated species interactions through quantitative aspects such as amount of food consumed by parasitized hosts as well as qualitative aspects such as composition of herbivore oral secretions (Poelman et al. 2011b; Tan et al. 2018). How parasitoids prolong herbivore development is understudied compared to arrestment of growth and we thus urge for studies that explore the physiological mechanisms that cause prolongation of host development. In addition, we require detailed understanding of how parasitoids manipulate their host environment beyond the traits that benefit the parasitoid itself. All traits that may influence how parasitoids influence plant-mediated species interactions should be considered. This includes to identify changes in host organs that may not be relevant to parasitoid development, but are crucial in the interface between parasitized herbivores and the food plant, such as salivary glands. A critical knowledge gap is which mechanisms in parasitoid host manipulation predict the outcome of plant-mediated species interactions and determine the net benefit of plants to recruit parasitoids as agents of indirect defence. Such knowledge could be profitable for the ongoing debate about the adaptive role of HIPVs in the attraction of parasitoids (Turlings and Erb 2018; Pearse et al. 2020). We thus pledge for revival of mechanistic, ecological and evolutionary studies on parasitoid host manipulation and emphasize that these studies should more prominently include parasitoids that prolong herbivore development.
